# New Edition of Burchard-Inglis Pathology

**Published:** 1915-07

**Authors:** 


NEW EDITION OF BURCHARD-INGLIS PATHOLOGY. The fifth edition of this standard work is before us, and like its predecessors, and owing to the faithful work of its editor and the publishers, it is up to date and much new material is presented. This is the most successful effort we know of where two subjects that are different are concordantly treated in the same text. While pathology and therapeutics are necessarily related they are so different fundamentally and scientifically that it is quite a problem to attempt a pedagogic treatment in the same text that will be interesting to the student, or that will not lead to more or less confusion in presentation to the class. This work comes nearer accomplishing this than any that has yet appeared. The present edition seeks to present the pathologic and the therapeutic in such close relations as to eliminate confusion as much as possible. The author has done well in accomplishing this without the continued repetition of therapeutic suggestions in the text that would seem almost inevitable. It is a most excellent book and does great credit to both editor and publishers. All progressive men will need this new work, and it certainly will be a great help to teachers. It is published by Lea and Febiger of Philadelphia.

